# Using preschoolers to improve caregivers' knowledge, attitude, and practices relating to biofortified crops: Evidence from a randomized nutrition education trial in Kenya

**DOI:** 10.1002/fsn3.2960

**Published:** 2022-07-22

**Authors:** Sylvester Okoth Ojwang’, Julius Juma Okello, David Jakinda Otieno, Rose Adhiambo Nyikal, Penina Ngusye Muoki

**Affiliations:** ^1^ Department of Agricultural Economics University of Nairobi Nairobi Kenya; ^2^ International Potato Centre Nairobi Kenya; ^3^ International Potato Centre Kampala Uganda; ^4^ Present address: HarvestPlus, c/o International Centre for Tropical Agriculture (CIAT) Nairobi Kenya

**Keywords:** biofortified crops, Kenya, knowledge, attitude, and practices, nudges, preschooler–caregiver pairs, vitamin A deficiency

## Abstract

This 2018 randomized controlled trial examined the role behavioral nudges can play in improving caregivers' knowledge, attitude, and practices (KAP) relating to biofortified orange‐fleshed sweetpotato (OFSP). The experiment involved 431 preschooler–caregiver pairs in 15 villages. The preschoolers were enrolled in public‐run Early Childhood Development and Education (ECDE) centers in the respective villages. Caregivers were first exposed to the routine OFSP promotion activities in the area – invited to cooking demonstration workshops and issued with free OFSP vines to plant. A baseline survey followed. Next, the 15 villages were randomized into four study groups (a control and three treatments). The interventions were deployed for 30 days as follows: Treatment 1 – preschoolers issued OFSP‐branded exercise books, class posters, and poems; Treatment 2 – caregivers received phone‐mediated text messages; and Treatment 3 – received the full suite of interventions. This study analyzed the endline and baseline data and finds that, in general, changes in KAP scores were negatively associated with control group (*p* = .005) and positively associated with Treatment 3 (*p* = .02). Specifically, Treatment 3 significantly increased caregivers' knowledge of OFSP production, consumption, and vitamin A. Treatment 2 significantly improved their attitude too. It concludes that an integrated complementary nutrition education approach targeting preschooler–caregiver pairs is more effective in increasing knowledge of cultivation and consumption of OFSP. It discusses the implications for the design of more effective nutrition programs targeting households with preschoolers to accelerate the fight against vitamin A deficiency (VAD).

## Graphical abstract

Integrated nutrition education approaches involving branding preschoolers’ learning materials with orange‐fleshed sweetpotato (OFSP) messages and sending these text messages to the caregivers’ mobile phones were tested in a village‐level cluster randomized control trial. These interventions showed significant positive effects on caregivers’ knowledge and attitude towards the OFSP. There is a great potential to improve the performance of nutrition programs involving biofortified crops by embracing preschoolers ‐ as agents of change ‐ and sending mobile phone‐mediated text reminders to the caregivers.

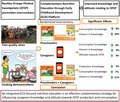

## 1 INTRODUCTION

Malnutrition is one of the major challenges in sub‐Saharan Africa where, for instance, the prevalence of undernourishment stands at 22.0% (FAO et al., 2020). Micronutrient deficiency is a relatively more persistent form of malnutrition as its prevalence decreases at a considerably lower pace even in the face of income growth (Barrett & Bevis, 2015). It is not easy to identify at early stages but can lead to irreversible long‐term effects on a child's cognitive capacity, physical ability, and economic productivity. Vitamin A deficiency (VAD) is among the most prevalent form of micronutrient deficiency, with young children and women of reproductive age being the most vulnerable (Nordin et al., 2013). It is the leading cause of acquired blindness, prevalence of morbidity among under‐5‐year‐olds and cases of low birth weight. In Kenya, the latest nationally representative data show that the prevalence of VAD and marginal VAD status are highest among preschool‐age children (6–59 months old) at 9.2% and 52.6%, respectively (Republic of Kenya, 2011).

As with other micronutrient deficiency cases, VAD is a result of low vitamin A intake due to lack of vitamin A‐rich food in the diet. Provitamin A biofortified staples, including orange‐fleshed sweetpotato (OFSP), can supply vitamin A‐rich diets, increase vitamin A intakes, and contribute significantly to avoiding VAD among rural farming households (Hotz, Loechl, de Brauw, et al., 2012; Low et al., 2017; Tanumihardjo et al., 2010; WHO, 2018). The orange color of the flesh of the roots indicates a high level of β‐carotene, a precursor of vitamin A, and is associated with higher acceptance among young vulnerable children. A regular serving of 125 grams of OFSP supplies the daily vitamin A needs of a young child, typically 300–1200 retinol activity equivalent units per 100 g (Hotz, Loechl, de Brauw, et al., 2012; Hotz, Loechl, Lubowa, et al., 2012).

Studies have confirmed that poverty and overall poor access to nutrient‐dense foods play a key role in poor nutrition (Siddiqui et al., 2020). Considerable literature also links malnutrition among rural households to poor attitudes and practices around nutritious food access and consumption behaviors (Acharya, 2018; FAO, 2011; Shikuku et al., 2019). However, there is continued evidence that active and well‐structured nutrition education that improves knowledge and attitude towards OFSP production, its nutritional benefits and utilization can help increase vitamin A intake among the vulnerable population (FAO, 2011; Kulwa et al., 2014; Mutiso et al., 2018; Suh & Chung, 2010; Tanumihardjo et al., 2010). Nutrition education has been used to influence changes in dietary practices (Jones & de Brauw, 2015; Kulwa et al., 2014; Low et al., 2017; USAID, 2014). Laurie et al. (2018) suggest that targeting the early childhood development (ECD) group with innovative information dissemination strategies can scale up the production and consumption of OFSP, especially in the rural settings. However, past nutrition education interventions rarely involve both the VAD high‐risk groups (young children and their caregivers, i.e., women of reproductive age), simultaneously. Despite the rise in school‐based interventions aimed at promoting utilization of nutritious food among school children, there is a limited understanding of the effects of nutrition education targeting preschoolers as change agents and their caregivers on the latter's knowledge of OFSP, attitude towards its production and use of recommended production practices.

The objective of this study was to assess the effects of complementary nutrition education interventions targeting preschoolers and their caregivers on knowledge, attitude, and practices (KAP) relating to OFSP among the households with preschoolers in Homa Bay County, Kenya. Specifically, this study sought to assess the effects of (i) preschooler‐focused nutrition education interventions on the caregivers' KAP relating to OFSP; (ii) caregiver‐targeted mobile‐phone‐mediated nutrition education reminders/messages on the caregivers' KAP relating to OFSP; and (iii) integrated complementary nutrition education interventions on the caregivers' KAP relating to OFSP.

To the best of our knowledge, no study has examined the role that preschooler‐focused nutrition education interventions can play in influencing households' KAP towards production and consumption of nutritionally enhanced biofortified food. Two studies, Nabugoomu et al. (2015) and Hummel et al. (2018), which involved school children or KAP, are noteworthy. Hummel et al. (2018) involved 2‐ to 5‐year‐old children in the evaluation of sensory attributes and acceptability of OFSP in Malawi. The children and their caretakers were both used as subjects, who tasted the boiled sweetpotato roots and responded to the questionnaire. Knowledge and attitude constructs were measured as part of potential contributors to the study outcomes. Nabugoomu et al. (2015) conducted a cross‐sectional survey of caregivers of 2–6‐year‐old children who had received nutrition education and/or training in OFSP production to assess their vitamin A‐related KAP and OFSP adoption among farming communities in Kampala, Uganda. They found a significant positive relationship between nutrition education and vitamin A‐related knowledge of the caregivers, their attitude towards health and child health practices, and consumption of vitamin A‐rich food. However, our study differs from both in two ways: (i) We focus on both the preschoolers and use of mobile phone messaging platform – as independent or integrated avenues – to influence caregivers' knowledge, attitude, and behavior towards OFSP. (ii) We use a systematic and rigorously designed approach in assessing the outcomes – a random assignment of participants to treatments and a two‐wave panel dataset to address identification problems.

## 2 MATERIALS AND METHODS

### 2.1 Study area and sampling

This study was conducted in Homa Bay County, a county with a very high prevalence of undernutrition in Kenya. Over one‐quarter of children under 5 years in the county are stunted (Bernstein & Wiesmann, 2018; Republic of Kenya, 2018). The county was, hence, targeted by a large project, which promoted OFSP production and utilization by farm households between 2015 and 2018.

Sampling proceeded as follows. Fifteen villages, where OFSP had not been introduced, were purposively selected in Ndhiwa and Rangwe subcounties. It was established from the larger OFSP promotion program that these villages were to receive OFSP promotion activities for the first time ever. In each selected village, one government‐run Early Childhood Development and Education (ECDE) center was selected for the study, thus 15 ECDE centers. Next, households with preschoolers enrolled at the selected ECDE centers were listed and caregivers of the enrolled children were invited to cooking demonstration workshops. The workshops were held at each of the ECDE centers in February 2018. Each caregiver was then given 200 cuttings (each 30 centimeters long) of free OFSP vines, in April 2018 to plant. These two activities (free vine dissemination and cooking demonstration workshops) define the routine OFSP promotion interventions targeting rural farmers that were carried out under multiple OFSP farmer outreach projects in western Kenya. In this study, the caregivers who participated in the cooking demonstrations and received the free vines formed the sampling frame (*n* = 721). A random sample of 431 preschooler–caregiver pairs was drawn for the experiment following McConnell and Vera‐Hernández (2015). The following parameters and assumptions were considered in estimating the sample size: unequal number of clusters in the four study groups (a total of 15 villages); low intracluster correlation of 0.01, an average of 26 households per cluster, and a 95% confidence level for a 20% increase in the outcome. This gave an estimated average of 98 households in each of the study groups. We also considered a 10% possible nonresponse rate following a recent study with pregnant women and mothers of young children in the study area (Mutiso, 2017). The preschooler–caregiver pairs were sampled proportionate to size of the population of children enrolled at the ECDE centers, thus more pairs were recruited in ECDE centers with more children and vice versa.

The ECDE centers in Kenya are meant to offer holistic preprimary education to young children aged 4 and 5 years old and are managed by the county governments (Republic of Kenya, 2013). However, it is common to find even 7‐year‐olds in the centers and it is reported that about 30% of pupils in western Kenya are enrolled in classes, which are typically lower than expected of their ages (Uwezo Kenya, 2014). Homa Bay county has 1183 ECDE centers with an enrollment rate of 76% (County Government of Homa Bay, 2018). In the context of this study, a preschooler is referred to as any child aged between 4 and 7 years who is enrolled in an ECDE center.

### 2.2 Experimental design and interventions

The study followed a randomized controlled trial design with preschooler–caregiver pairs as participants. The sample was randomly assigned into four study groups (one control and three treatment arms) at village level – all preschooler–caregiver pairs in a given village were assigned into one study group. The control group participated only in the routine OFSP promotion activities – cooking demonstration workshop and got free vines. Conversely, the treatment groups in addition received complementary nutrition education interventions via three distinct approaches for 30 days in September and October 2018. We describe the three treatments below.

#### 2.2.1 Preschooler treatment (PT)

The PT group received OFSP messages through branded exercise books, poems, and classroom‐mounted posters that targeted the preschoolers only. The book covers and posters had pictorial illustrations of OFSP and a brief description of its health benefits. The text was written in *Dholuo* – the local language (see Pictures S1 and S2). Five OFSP‐related messages were communicated through these materials, hence five different categories of books and classroom posters. Each preschooler in this group received an exercise book. Their class teachers displayed the five posters in the classroom and read them out each school day. The teachers also recited the poem with the preschoolers each school day. The goal was to nudge the preschoolers into taking the messages home and persuade their caregivers to grow and consume OFSP.

#### 2.2.2 Caregiver treatment (CT)

In this treatment group, each caregiver received one short text message corresponding to those in exercise books, poem, and poster on their mobile phones every day for 30 days. Each of the seven messages in PT group's exercise books was sent once per week to caregivers – thus seven messages in 7 days. This was repeated four times over the 30‐day intervention period. The messages and built‐in reminders were intended to increase knowledge and nudge caregivers into developing positive attitude towards OFSP and growing it.

#### 2.2.3 Integrated treatment (IT)

**FIGURE 1:**
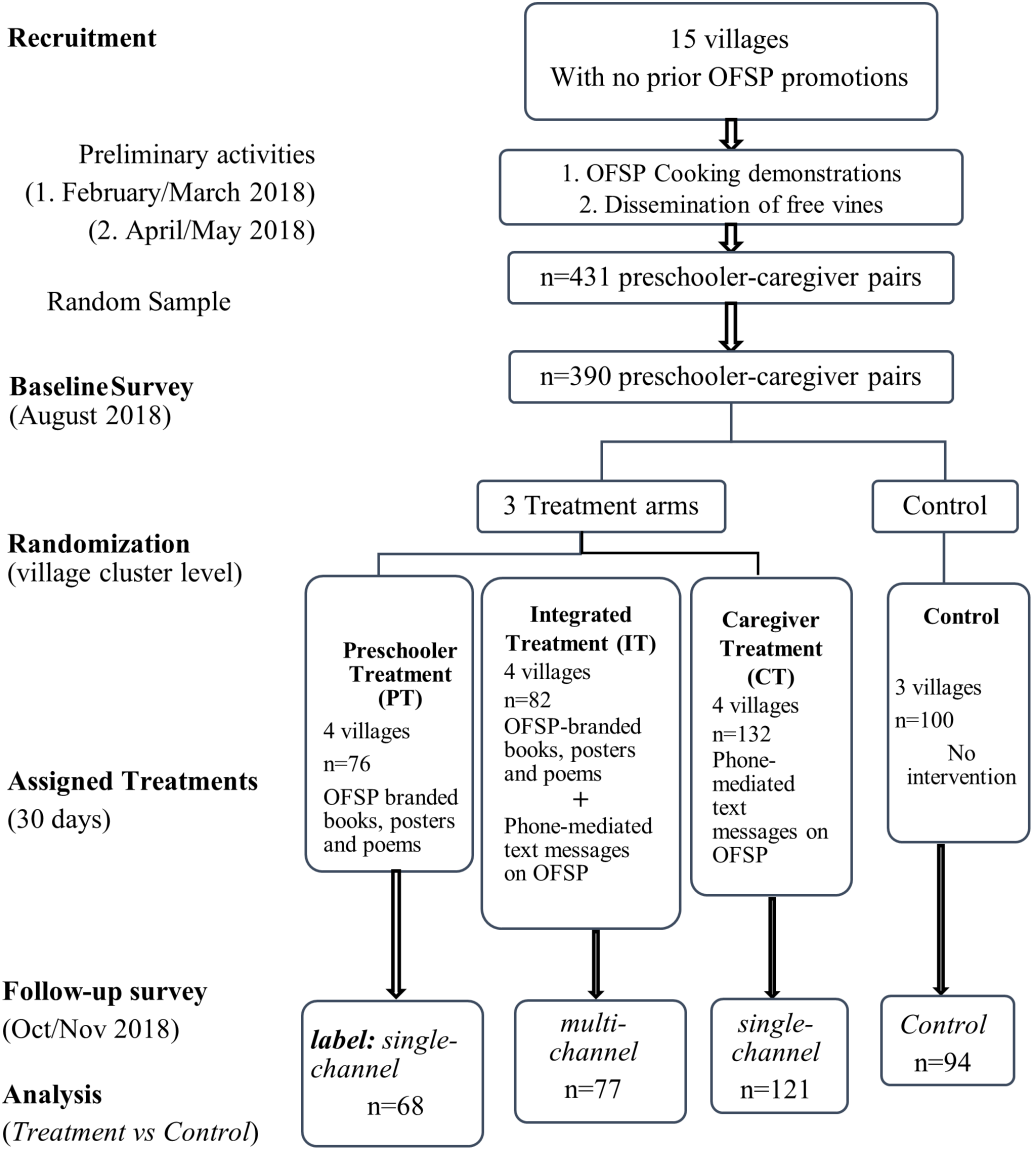
A CONSORT diagram of the study procedure

The IT group received both interventions received by the PT and CT groups, concurrently. In essence, for each household in this group, the mobile‐phone‐mediated messages on OFSP were sent to the caregiver for 30 days, the preschooler got an OFSP‐branded exercise book, had the OFSP posters mounted in their classroom and read out to them, and recited a poem with messages on nutritional benefits of OFSP on each school day. Figure 1 presents a graphical representation of the study procedure.

### 2.3 Data collection

Data were collected from caregivers using individually administered pretested and validated questionnaires at baseline and the end of the intervention period. The interviews were conducted in August 2018 (baseline) and October–November (follow‐up). Verbal informed consent was obtained from all the caregivers before proceeding with the interviews. A total of 390 and 360 complete interviews were conducted during the baseline and follow‐up surveys, respectively. The 7% attrition resulted from school absenteeism by preschoolers' and/or caregivers' refusal to participate in the follow‐up survey. However, there were no significant differences in the attrition rates between the study groups (Pearson's chi‐square = 1.6331; *p*‐value = .652).

The questionnaires captured, among others, data on the caregivers' sociodemographics, KAP, caregiver engagement with the interventions, and other institutional factors around the production and utilization of OFSP.

### 2.4 Measurement of caregivers' KAP constructs

Following Marias and Glasauer (2014) and Trakman et al. (2017), the dimensionality and reliability of various KAP survey segments were assessed and confirmed before the computation of the following dependent variables.

#### 2.4.1 Knowledge

An exploratory factor analysis of the baseline KAP data assessed the caregivers' knowledge of OFSP using a set of 19 items. These loaded into three factors: OFSP production knowledge (9 items), consumption knowledge (4 items), and vitamin A knowledge (6 items). For both the positively and negatively framed production and consumption knowledge statements, codes 0, 1, and 2 were entered for incorrect, correct, and “Don't know” responses, respectively. The “Don't know” responses were treated as unanswered questions to avoid penalizing respondents for their nonresponse to the items (Denman et al., 2018; Lietz, 2010). The final measure of the caregivers' OFSP production, consumption, or vitamin A knowledge was computed as a ratio of an individual's total correct scores to the total possible scores from all the responses. This gave a ratio scale of values 0 to 1. A value closer to 0 (1) implied a very poor (very good) knowledge level.

#### 2.4.2 Attitude

The caregivers responded to 11 items portraying an individual's general attitude towards OFSP. The items were measured on a 5‐point Likert scale ranging from “Strongly Disagree” to “Strongly Agree.” Positive and negative statements were intermixed and coding for the latter reversed as recommended by the literature (Lagerkvist et al., 2015; Mutiso et al., 2018). A Cronbach's alpha statistics of 0.78 (for 11 items) indicated that the set of items was reliable (Trakman et al., 2017). From the codes, the minimum and maximum total scores were 11 and 55, respectively. The attitude scores were also computed as a ratio ranging from 0 to 1. A value close to 0 (1) implied a highly negative (positive) attitude towards OFSP.

#### 2.4.3 Practices

A set of 15 items were used to assess the extent of caregivers' use of recommended OFSP production practices. Behaviors associated with OFSP consumption were excluded from the analysis since they were assessed only once – during the follow‐up survey. The items were measured using a dichotomous scale with “no”/“yes” responses coded as 0/1. The “Kuder‐Richardson Formula 20” test statistics of 0.73 against a threshold of 0.70 confirmed the reliability of the items in measuring the construct (Trakman et al., 2017). Total scores by an individual were divided by the total possible scores (15) to produce a ratio scale ranging between 0 and 1. A value closer to 0 (1) implied a highly poor (good) practice level.

### 2.5 Data analysis

#### 2.5.1 Exploratory analysis

All data were entered and analyzed using Stata version 14.2. After the descriptive statistics, an orthogonality test was done to ascertain whether randomization of the assignments was independent of the sociodemographic characteristics of the study groups. Baseline differences in the sociodemographic and institutional factors across the study groups were assessed using the two‐way analysis of variance (ANOVA) and Kruskal–Wallis tests for normally and non‐normally distributed variables, respectively. We also used the Bonferroni adjustment method for multiple pairwise comparisons of differences of the variables across the study groups (using Stata command *dunntest*) (Dinno, 2015). Additionally, Wilcoxon signed‐rank tests were used to evaluate within‐group (each study group) differences in the caregivers' KAP scores between baseline and follow‐up data. Between‐group differences in the mean changes in KAP scores (follow‐up score minus baseline score) were also evaluated using Kruskal–Wallis tests and the Bonferroni correction method.

#### 2.5.2 Empirical analysis

##### Treatment effects estimation

Our primary outcomes of interest, changes (follow‐up minus baseline) in the relative KAP scores, appear on a continuous scale with values from −1 to 1. Following McCullagh (2019), we employed a generalized linear model (GLM) specified as presented in Equation (1) below to model the changes in the KAP scores due to the treatments while accounting for the controls too.

(1) $$Y_{\mathrm{ij}}=\beta_{0}+\beta_{1}{\mathrm{PT}}_{\mathrm{ij}}+\beta_{2}{\mathrm{IT}}_{\mathrm{ij}}+\beta_{3}{\mathrm{CT}}_{\mathrm{ij}}+\beta_{4}{\mathrm{KAP}}_{\mathrm{ijt}=0}+X_{\mathrm{ij}}^{\prime }\beta_{n}+\varepsilon_{\mathrm{ij}}$$

where Y_*ij*_ is the change in the relative KAP scores for individual *i* in village *j*; and *PT*, *IT*, and *CT* are the random treatment group assignment variables coded as a dummy (1 for the given intervention group and 0 for being in the control group). The main parameters of interest *β*_1_, *β*_2_, and *β*_3_ represent the estimated average treatment effects of the interventions on the caregiver's KAP scores. *KAP*_*t*=0_ is the relative score of the respective KAP constructs at baseline for individual *i*. The vector *X'* contains individual‐level, household‐level, and institutional variables that relate to psychosocial constructs on food items. The random error term is notated as $\varepsilon_{i}$.

Furthermore, we employed the wild cluster *bootstrap‐t* procedure to estimate the *p*‐values for correct testing of the hypotheses – a solution to the problem of few clusters (because we have only 15 unbalanced clusters divided by 4 study groups) (Cameron & Miller, 2015; Duflo et al., 2008; Menger, 2017). In essence, we used the generalized linear regression with wild cluster bootstrapping, to estimate changes in individuals' relative KAP scores due to their assignment into different intervention groups relative to the control group while controlling for their baseline KAP scores and other covariates. Caregivers' age and household expenditure variables were excluded from the regression table as they showed very insignificant contribution to the estimation of the outcomes as shown by insignificant and very low coefficient values, and no significant change in the model fitness scores.

## 3 RESULTS AND DISCUSSION

### 3.1 Sample characteristics

As shown in Table 1, overall, the average caregiver was 36(±12) years old. However, the IT (*n* = 77) group was, on average, significantly younger than the rest of the groups. Perumal et al. (2013) observed that the age of antenatal mothers is positively related to their knowledge of and attitude towards child health and nutrition. Arguably, the level of an individual's KAP about a food crop may be related to their experience in farming and family healthcare, and their ages. As expected, female caregivers dominated (89%) the study, with a majority of them being the mothers of the preschoolers. This was expected, given that sweetpotato is traditionally regarded as a woman's crop and women tend to have more influence on the children's food preparation than their male counterparts (Low et al., 2017; Opiyo et al., 2010).

**TABLE 1:** Distribution of the sociodemographic variables across the study groups

Variables	Total sample (*n* = 360)	PT (*n* = 68)	CT (*n* = 121)	IT (*n* = 77)	Control (*n* = 94)	*p*‐value
		Books + posters	SMS only	Books + posters + SMS		
	Mean (SD)	Mean (SD)	Mean (SD)	Mean (SD)	Mean (SD)	
Child's age (years)	5.74 (1.10)	5.69^a^ (1.11)	5.6^a^ (1.14)	5.73^a^ (1.12)	5.94^a^ (1.02)	.226
Caregiver's age (years)	36.01(12.12)	35.66^a^ (11.27)	37.10^a^ (13.02)	31.47^b^ (9.17)	38.56^a^ (12.76)	.001
Caregiver's education level (years)	7.20 (2.73)	7.01^a^ (2.49)	7.36^a^ (2.75)	7.64^a^ (1.94)	6.77^a^ (3.34)	.202
Household size (scale)	6.37 (2.11)	6.63^a^ (2.08)	6.17^a^ (1.95)	6.12^a^ (1.82)	6.64^a^ (2.51)	.188
HH's monthly expenditure (USD)	71.39(75.92)	68.98^b^ (59.70)	67.91^b^ (75.41)	51.24^b^ (35.21)	94.12^a^ (102.25)	.003
Distance to CHV (walking minutes)	15.21(15.77)	9.56^a^ (12.27)	18.53^a^ (17.03)	13.81^b^ (11.51)	16.17^a^ (18.12)	.002
OFSP farm size in 1st season (m^2^)	41.53(52.19)	44.89^ab^ (55.61)	35.94^a^ (41.51)	37.48^b^ (54.45)	44.25^a^ (61.17)	.056
Caregiver's sex (% female)	89	93^a^	88^a^	91^a^	86^a^	.580
Households with under‐5‐year olds (%)	76	74^a^	78^a^	79^a^	73^a^	.605
Household head's sex (% female)	17	16^a^	19^a^	12^a^	17^a^	.926
Married (yes/otherwise) % married	83	82^a^	80^a^	87^a^	84^a^	.646
Member of a farmer group (0/1) %	32	29^a^	29^a^	35^a^	36^a^	.615
HH grew white/yellow SP (%)	67	60^a^	69^a^	65^a^	72^a^	.411
Grew OFSP in 1st season (%)	53	57^a^	31^b^	47^ab^	81^c^	.000

*Note*: (1) Standard deviations (SDs) in parentheses. Last column displays results (*p*‐values) from two‐way analysis of variance (ANOVA) and Kruskal–Wallis tests for differences between the four study groups. (2) Superscript letters present results for pairwise test of differences in means between two study groups after Bonferroni correction method for multiple pairwise comparisons of sample means. Matching superscripts, **aa** or **bb**, imply no significant differences between the study groups by the given variable, while nonmatching superscripts, **ab**, imply otherwise.

Abbreviations: CHV, community health volunteer; Control, control group; CT, caregiver treatment group; HH, household; IT, integrated treatment group; PT, preschooler treatment group.

Only a third of the entire sample participated in farmer groups. This finding points to the limited farmer‐based network for sharing knowledge about different production and nutrition improvement technologies on OFSP and other nutrient‐rich food crops. However, they reported an average travel time of 15 walking minutes from their homes to the closest community health volunteers (CHVs). Okello et al. (2019) document how CHVs have previously been engaged by agriculture–nutrition‐sensitive projects to promote OFSP. However, the current study area had not been reached with such projects.

A majority (67%) of the sample grew the white‐fleshed and yellow‐fleshed sweetpotato varieties with no significant differences across the study groups. On the other hand, about one‐half of the total sample harvested OFSP in the first season. However, a significantly higher percentage (81%) of the control group grew the OFSP than in the other study groups. This was mainly due to the differences in rainfall patterns during planting season, which largely favored one village in the control group.

The preschooler's average age was 6 years (and ranged 4–7 years); hence, a majority could relay information received in school to the caregivers. Children were used in the study as change agents, and their effectiveness may be related to their ages. Hence, the preschoolers who participated in the group could potentially serve the intended role.

The ECDE centers are indeed a good avenue for reaching households with VAD high‐risk groups. About 76% and 75% of the households had under‐5‐year‐old children and female caregivers of reproductive ages (15–49 years), respectively. In the latter case, the remaining one‐quarter of the households had caregivers who form an active social environment to the children (the fathers, grandmothers, and aunts) and should be actively integrated into agriculture–nutrition promotion interventions for optimal success (Mutiso et al., 2018).

### 3.2 Distribution of KAP scores across study groups and time

As shown in Table 2, only caregivers' practices relating to OFSP significantly differed across the study groups at both the baseline and follow‐up levels. The OFSP consumption and vitamin A knowledge only differed between the study groups at follow‐up level, while the production knowledge and attitude constructs had no statistically significant differences at both the baseline and follow‐up levels. Overall, all the study groups had significant improvements in their vitamin A knowledge and attitude towards OFSP after the intervention period (*p* < .05). However, only the caregivers in the IT recorded significant increase in their OFSP production and consumption knowledge. None of the study groups recorded a statistically significant improvement in their level of practice regarding OFSP. This could be because of the relatively short period between baseline and follow‐up. Caregivers may not have had enough time to learn about OFSP and implement recommended practices.

**TABLE 2:** Mean of caregivers' knowledge, attitude, and practices (KAP) scores before and after the intervention across the study groups

	PT (*n* = 68)	CT (*n* = 121)	IT (*n* = 77)	Control (*n* = 94)	*p‐*value^†^
	Mean (SD)	Mean (SD)	Mean (SD)	Mean (SD)	
Production knowledge (B)	0.661^a^ (0.188)	0.671^a^ (0.179)	0.656^a^ (0.188)	0.670^a^ (0.182)	.912
Production knowledge (A)	0.673^ab^ (0.176)	0.704^ab^ (0.153)	0.743^a^ (0.131)	0.684^b^ (0.162)	.073
Δ in production knowledge	0.011^a^ (0.254)	0.032^a^ (0.235)	0.098^a^ (0.184)	0.014^a^ (0.235)	.106
***p‐*value** ^‡^	.608	.201	<.001	.619	
Consumption knowledge (B)	0.922^a^ (0.167)	0.946^a^ (0.119)	0.929^a^ (0.129)	0.938^a^ (0.137)	.724
Consumption knowledge (A)	0.966^a^ (0.106)	0.958^a^ (0.094)	0.971^a^ (0.081)	0.916^b^ (0.130)	.001
Δ in consumption knowledge	0.038^a^ (0.196)	0.015^ab^ (0.152)	0.041^a^ (0.155)	‐0.021^b^ (0.2)	.040
***p‐*value** ^**‡**^	.116	.396	.035	.142	
Vitamin A knowledge (B)	0.629^a^ (0.330)	0.652^a^ (0.304)	0.647^a^ (0.305)	0.687^a^ (0.291)	.625
Vitamin A knowledge (A)	0.872^ab^ (0.206)	0.861^ab^ (0.185)	0.887^a^ (0.194)	0.805^b^ (0.289)	.024
Δ in vitamin a knowledge	0.243^ab^ (0.337)	0.23^ab^ (0.319)	0.240^a^ (0.347)	0.118^b^ (0.373)	.080
***p‐*value** ^**‡**^	<.001	<.001	<.001	<.001	
Attitude towards OFSP (B)	0.609^a^ (0.110)	0.636^a^ (0.109)	0.623^a^ (0.092)	0.617^a^ (0.109)	.350
Attitude towards OFSP (A)	0.690^a^ (0.111)	0.699^a^ (0.124)	0.683^a^ (0.111)	0.667^a^ (0.103)	.130
Δ in attitude level	0.081^a^ (0.154)	0.063^a^ (0.144)	0.060^a^ (0.145)	0.050^a^ (0.140)	.535
***p‐*value** ^**‡**^	<.001	<.001	<.001	.002	
Practices around OFSP (B)	0.286^ab^ (0.162)	0.247^b^ (0.149)	0.273^b^ (0.180)	0.352^a^ (0.212)	.003
Practices around OFSP (A)	0.325^ab^ (0.231)	0.256^b^ (0.159)	0.302^b^ (0.220)	0.351^a^ (0.164)	<.001
Δ in practice level	0.039^a^ (0.268)	0.009^a^ (0.207)	0.030^a^ (0.233)	‐0.001^a^ (0.239)	.816
***p‐*value** ^**‡**^	.326	.934	.320	.616	

*Note*: (1) KAP score values range from 0 to 1. (2) (B) = Before intervention, and (A) = After intervention. (3) Change (Δ) in KAP scores range from −1 to 1 and imply follow‐up minus baseline scores. (4) ^**†**^ *p*‐values from Kruskal–Wallis H tests for differences between the four study groups. (5) ^‡^ *p*‐values from Wilcoxon's signed‐rank tests for differences within groups (differences in mean KAP scores between before and after intervention). (6) Bold *p*‐values imply statistically significant differences at a 5% level of significance. (7) Matching superscripts, **aa** or **bb**, imply no significant differences between the study groups by the given variable, while nonmatching superscripts, **ab**, imply otherwise.

Abbreviations: Control, control group; CT, caregiver treatment group; IT, integrated treatment group; PT, preschooler treatment group.

**FIGURE 2:**
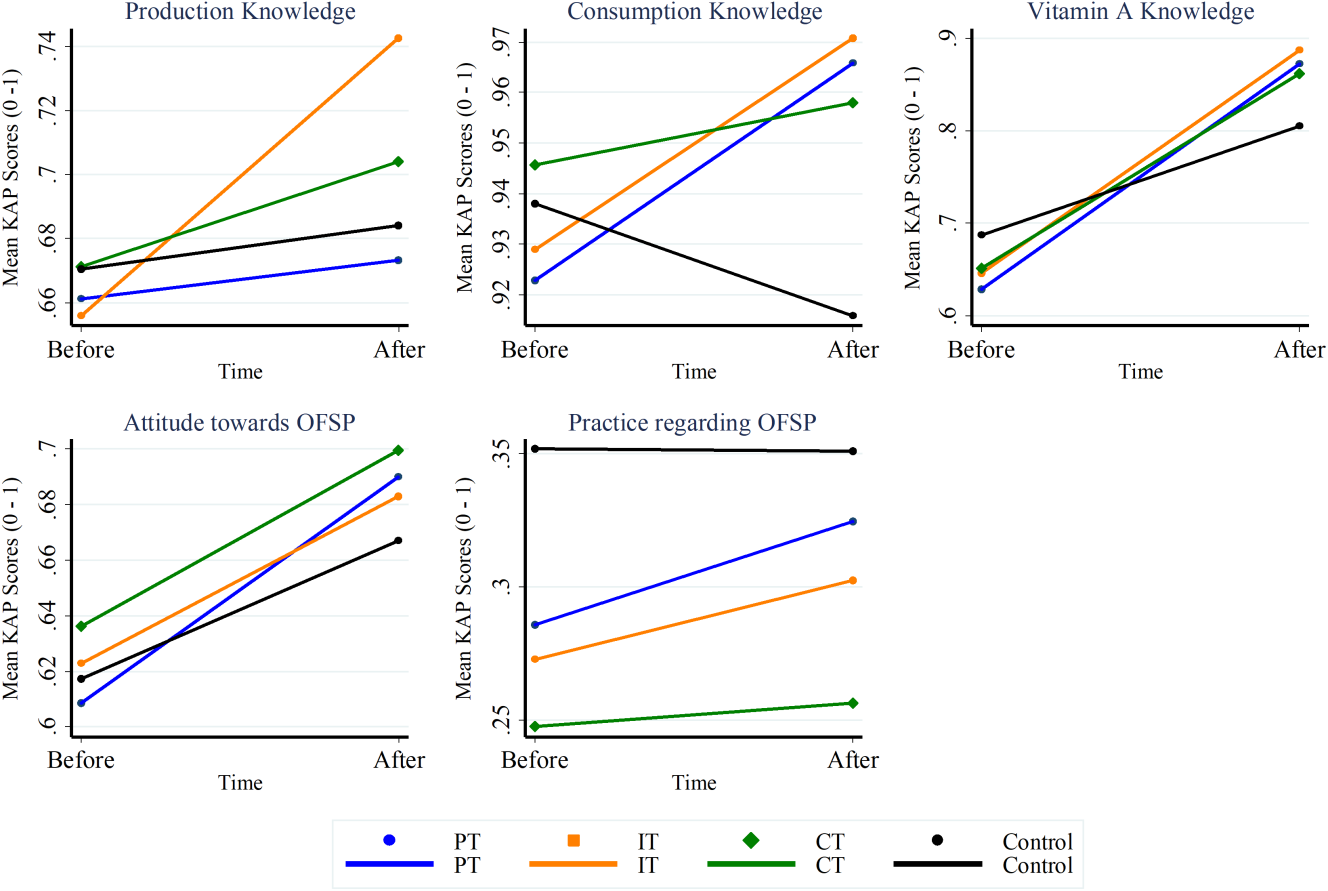
Trends of mean KAP scores before and after the intervention by the study group. The comparison of the average KAP scores per study group before and after the intervention. Control, control group; CT, caregiver treatment group; IT, integrated treatment group; KAP, knowledge, attitude, and practices; PT, preschooler treatment group

Figure 2 below illustrates how the treatment groups scored on the different KAP constructs before and after the intervention. At baseline, the caregivers recorded a good mean score (>0.8) in only the OFSP consumption knowledge construct; average mean scores (slightly above 0.6) in the production knowledge, vitamin A knowledge, and attitude constructs; and poor mean scores (<0.5) in the practices towards OFSP construct. The good consumption knowledge scores at baseline can be attributed to the caregivers' participation in the OFSP cooking demonstration sessions before the launch of the interventions.

Ribeiro et al. (2015) noted that cooking styles have varied effects on bio‐accessibility of β‐carotene in provitamin A‐rich foods. Besides reinforcing knowledge on appropriate cooking methods, cooking demos are also intended to ensure cultural acceptability of the food in the diets. Also, the poor performance by the caregivers on the practice construct can partly be attributed to the fact that this was the first time that they engaged with the OFSP.

Postintervention, there were increased mean scores in the production knowledge constructs across the study groups. However, the changes in the IT treatment group were more significant than for the rest of the groups. Also, all the treatment groups recorded improvements in their consumption knowledge mean scores. This could imply the possibility of erosion of knowledge of the nutritious crops in the absence of continuous nutrition education and reminders. In addition, all the groups recorded increases in their vitamin A knowledge and attitude towards OFSP. Overall, the variation in the behavior of the treatment groups against the control groups suggests the need to further systematically explore how the different interventions worked in follow‐up studies.

### 3.3 Mean changes in KAP scores

Every third and fourth row of Table 2 presents the distribution of the mean changes in the respective KAP scores across the study groups and the significance levels, respectively. The results show that, over time, an individual's knowledge, attitude, and practices towards OFSP are destined to change. However, a positive change is desirable to leverage the pathways to improved nutritional status among households targeted by agriculture–nutrition‐sensitive interventions. All the intervention groups recorded positive mean changes in the KAP scores, while the control groups recorded a mix of positive and negative mean changes although some changes were not statistically significant. The Spearman correlation results indicate that, overall, change in the KAP scores was negatively associated with an individual's assignment into a control group (Spearman's rho = −0.143, *p =* .005), and positively associated with their assignment into the IT group (Spearman's rho = 0.129, *p =* .015). In terms of the magnitude of change, only the mean changes in OFSP consumption knowledge and vitamin A knowledge scores were significantly higher in the PT and IT groups than in the control group. However, we acknowledge that these associations do not sufficiently imply causations. Thus, the effectiveness of these reported relationships in informing substantive policy recommendations is limited. We, therefore, explore the cause‐and‐effects in section 3.4 below.

### 3.4 Treatment effects

Table 3 presents the estimates of the generalized linear models of each of the mean changes in KAP scores. After controlling for the differences in sociodemographics and the respective baseline KAP scores, nutrition education through the PT approach significantly improved the caregivers' knowledge of OFSP consumption and vitamin A benefits. The IT approach, on the other hand, improved the caregivers' OFSP production knowledge, consumption knowledge, and the vitamin A knowledge. Similarly, the CT approach improved the caregivers' consumption knowledge, vitamin A knowledge, and attitude towards OFSP. None of the approaches caused statistically significant improvements in the caregivers' practice relating to OFSP production and consumption. The findings are similar to those of a study in Uganda (Nabugoomu et al., 2015), which showed that nutrition education improved the caregivers' knowledge related to vitamin A and OFSP as a source of vitamin A and their attitude towards child healthcare practices. However, the study focused on an urban and peri‐urban farming setting and had a different nutrition education approach.

**TABLE 3:** Generalized linear regression models for mean change in orange‐fleshed sweetpotato (OFSP) knowledge, attitude, and practice (KAP) scores among the caregivers of preschool children in the study sample^†^

	Production knowledge	Consumption knowledge	Vitamin A knowledge	Attitude	Practices
*Treatment variables*					
Preschooler treatment (PT)	−0.011 (−0.64)	0.056*** (4.24)	0.096* (1.66)	0.025 (1.24)	0.011 (0.31)
Caregiver treatment (CT)	0.021 (1.24)	0.063*** (11.51)	0.094* (1.84)	0.038** (2.67)	−0.011 (−0.33)
Integrated treatment (IT)	0.054** (2.54)	0.068*** (5.55)	0.106* (1.75)	0.012 (0.95)	0.007 (0.14)
*Controls* ^†^	Yes	Yes	Yes	Yes	Yes
Constant	0.668*** (11.25)	0.929*** (12.55)	0.494*** (3.83)	0.536*** (7.95)	0.219** (2.88)
Observations	355	349	360	360	360
Wald *χ* ^2^	1484.2	6112.0	12,202.8	792.8	4995.2
*p*‐value > |*χ* ^2^|	<.001	<.001	.000	<.001	.000
*R* ^2^	0.558	0.668	0.626	0.418	0.476

*Note*: *t* statistics in parentheses; wild cluster bootstrap‐*t* procedure used to adjust the p‐values (not presented); *p < .10, **p < .05, ***p < .001. ^†^The five models testing for the effect of intervention assignments were adjusted to control for the baseline scores for the respective KAP categories and the variables, which were found to be significantly different between the study groups at baseline. See Table S1 for the full model results. Production knowledge and consumption knowledge models have samples short of 360 due to missing data – five and eleven respondents failed to respond to all the items in the respective construct measures at either baseline or follow‐up survey.

The performance of the interventions suggests significant improvements in the different dimensions of nutritional knowledge relating to OFSP if an integrated nutrition education intervention is used (i.e., the IT intervention). Comparatively, the CT intervention performed better than the PT intervention in improving the knowledge constructs. Given the fact that, overall, the entire sample had very poor attitude scores, a deliberate focus on improving their attitude scores is more critical. The results show that the phone‐mediated messages and reminders are potentially more effective means of influencing the caregivers' attitude towards OFSP. Furthermore, as mediators of behavior change, improvements in both knowledge and attitude may have trickle‐down effects on the availability of the OFSP in the household menus and intake of vitamin A.

These results are broadly in line with the propositions of the social cognitive theory and findings of Mutiso (2017), which show evidence for the use of a multicomponent approach when intervening with children to enhance the effectiveness of information reception, retention, and transfer to others, including caregivers. The daily transmission of the messages to the households via phone‐based text messages enhanced the response of caregivers. Indeed, the CT treatment had a greater effect on caregivers' attitude towards OFSP compared to the control group.

Furthermore, with the positive results of phone‐mediated messages on improving the caregivers' overall knowledge and attitude towards OFSP in the CT approach, the study underscores the role ICT can play in influencing knowledge, attitude, and practices relating to biofortified crops, their acceptance and utilization in the rural households.

The findings also contribute to the literature on the effectiveness of information and communications technology (ICT)‐integrated nutrition education in improving the diets of rural farming households (Webb, 2013). The CT approach significantly increased the knowledge of consumption and vitamin A, as well as attitude but had no effect on production knowledge or practice. On the other hand, the preschooler targeting was only effective in improving knowledge. The latter finding is in line with our a priori expectations because preschoolers are not involved in cooking and production practices, thus their influence is limited to providing information awareness.

## 4 CONCLUSION AND IMPLICATIONS

This study examined the effects of nutrition education interventions targeting preschoolers, as change agents, and their caregivers with nudges designed to influence caregivers' knowledge, attitude, and practices relating to OFSP production and consumption. The participants – preschooler–caregiver pairs – were randomly assigned into treatments/interventions promoting OFSP production and consumption. The study finds that participants who received all the interventions had significantly higher knowledge scores than those who received none of the interventions. It also finds that none of the interventions had a significant effect on farmer practices relating to OFSP production and consumption.

Based on the findings, we conclude that an integrated nutrition education intervention involving preschoolers and phone‐mediated text messages to the caregivers is an effective strategy for influencing caregivers' knowledge and attitude. We further conclude that interventions targeting preschoolers only are more effective in improving knowledge of caregivers, while those that specifically target caregivers through mobile‐phone‐mediated messaging are effective on caregivers' knowledge of and attitude towards OFSP.

The study findings suggest that preschoolers and their learning materials can significantly nudge the caregivers and influence their knowledge of OFSP production, consumption, and nutritional content. This implies that government and basic education providers could effectively scale up the impact of nutrition programs by strategically integrating nutrition education messages on the learning materials of children enrolled in the ECDE centers.

The findings also imply that mobile‐phone‐mediated text messaging can be used effectively to influence the caregivers' knowledge and attitude towards OFSP. The advantages of using mobile‐phone‐mediated education and extension over face‐to‐face have been widely documented in research and development literature and include, among others, the low cost. However, this is not yet established for this approach targeting improved psychosocial constructs among caregivers. Overall, a cost‐effectiveness analysis of the three approaches will be a significant contribution to knowledge and nutrition policy suggestions and should be considered for further research.

## CONFLICT OF INTEREST

The authors declare that they do not have any conflict of interest.

## ETHICAL REVIEW

This study followed the ethical guidelines in the Declaration of Helsinki. It was authorized by the Homabay County Early Childhood Development Education Office under REF: HBC/EDUC&ICT/PTN/VOL. 1/4/1/8/034 of July 30, 2018. It was also approved by the University of Nairobi's Graduate School under Ref: A56/89965/2016.

## INFORMED CONSENT

The caregivers gave written informed and voluntary consents on behalf of themselves and their preschoolers.

## Supporting information


**Appendix S1:** Supporting information

## Data Availability

The data that support the findings of this study are available from the corresponding author upon request.
