# Supplementary material for: Using preschoolers to improve caregivers' knowledge, attitude, and practices relating to biofortified crops: Evidence from a randomized nutrition education trial in Kenya
Source: Food Sci Nutr. 2022 Jul 22;10(11):3627–37. doi: 10.1002/fsn3.2960 (PMC9632203; doi:10.1002/fsn3.2960)
Supplement: Supplementary file 1 — Appendix S1: Supporting information [file FSN3-10-3627-s001.docx]

# Supplemental Data

# Supplemental Picture 1. A sample Cover Page of OFSP-branded Exercise Books


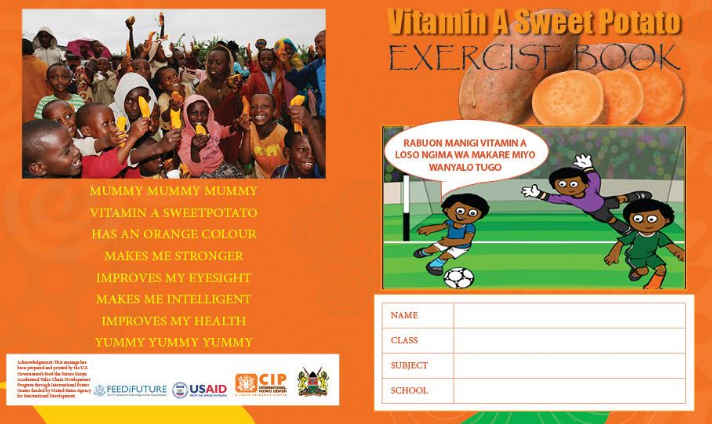


# Supplemental Picture 2. Sample OFSP-branded Class Posters


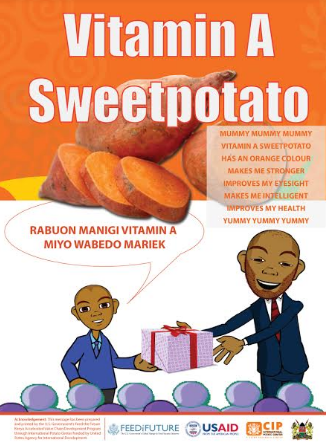


**Supplemental Table 1.** Generalized Linear Regression Models for Mean Change in OFSP Knowledge, Attitude, and Practice Scores among the Caregivers of Preschool Children in the Study Sample^†^

|  | **Production Knowledge** | **Consumption Knowledge** | **Vitamin A Knowledge** | **Attitude** | **Practices** |
| --- | --- | --- | --- | --- | --- |
| ***Treatment variables*** |  |  |  |  |  |
| Preschooler Treatment (PT) | -0.011 (-0.64) | 0.056^***^ (4.24) | 0.096^*^ (1.66) | 0.025 (1.24) | 0.011 (0.31) |
| Integrated Treatment (IT) | 0.054^**^ (2.54) | 0.068^***^ (5.55) | 0.106^*^ (1.75) | 0.012 (0.95) | 0.007 (0.14) |
| Caregiver Treatment (CT) | 0.021 (1.24) | 0.063^***^ (11.51) | 0.094^*^ (1.84) | 0.038^**^ (2.67) | -0.011 (-0.33) |
| ***Controls*^†^** |  |  |  |  |  |
| Production Knowledge at baseline (kp0) | -0.929^***^ (-22.57) | - | - | - | - |
| Consumption Knowledge at baseline (kc0) | - | -1.037^***^ (-35.96) | - | - | - |
| Vitamin A Knowledge at baseline (VA0) | - | - | -0.871^***^ (-17.37) | - | - |
| Attitude towards OFSP at baseline (att0) | - | - | - | -0.871^***^ (-11.58) | - |
| Practice level at baseline (pract0) | -0.043 (-0.84) | 0.075^**^ (2.59) | 0.235^***^ (5.21) | 0.029 (0.68) | -0.898^***^ (-19.25) |
| Distance to Community Health Volunteers (*square root)* | 0.002 (0.08) | -0.005 (-1.59) | 0.003 (0.50) | -0.001 (-0.34) | -0.004 (-1.47) |
| Grew OFSP to maturity (1^st^ season) | 0.017 (0.95) | 0.016 (1.25) | 0.025^*^ (1.86) | 0.018 (1.23) | 0.159^***^ (6.19) |
| Child Dietary Diversity Score | -0.008 (-1.16) | 0.001 (0.15) | -0.004 (-0.53) | 0.0005 (0.08) | -0.002 (-0.28) |
| Aware of vine multiplier in the village | 0.040 (1.49) | -0.0342 (-1.18) | 0.00393 (0.11) | 0.0200 (0.95) | 0.079 (1.52) |
| Distance to market (*square root)* | 0.003 (0.63) | 0.005^**^ (2.10) | -0.0025 (-0.40) | 0.0039 (1.28) | -0.002 (-0.50) |
| Household Food Insecurity (Access) Scale | -0.001 (-0.43) | -0.000 (-0.04) | 0.002 (0.88) | -0.003^**^ (-2.98) | 0.000 (0.03) |
| Caregiver’s Age (Years) | -0.031 (-0.09) | -0.179 (-0.81) | 0.679^*^ (1.83) | 0.277 (1.21) | -0.058 (-0.16) |
| Constant | 0.668^***^ (11.25) | 0.929^***^ (12.55) | 0.494^***^ (3.83) | 0.536^***^ (7.95) | 0.219^**^ (2.88) |
| Observations | 355 | 349 | 360 | 360 | 360 |
| Wald Chi^2^ | 1484.2 | 6112.0 | 12202.8 | 792.8 | 4995.2 |
| *p*-value > \|*χ^2^*\| | <0.001 | <0.001 | <0.001 | <0.001 | <0.001 |
| *R^2^* | 0.558 | 0.668 | 0.626 | 0.418 | 0.476 |

Notes: Values are parameter estimates; *t* statistics in parentheses; wild cluster bootstrap-t procedure used to adjust the p-values (not presented); ^*^*p*< 0.10, ^**^*p*< 0.05, ^***^*p*< 0.001. ^†^The five models testing for the effect of intervention assignments were adjusted to control for the baseline scores for the respective KAP categories and the variables, which were found significantly different between the study groups at baseline. Production knowledge and consumption knowledge models have samples short of 360 due to missing data – five and eleven respondents failed to respond to all the items in the respective construct measures at either baseline or follow-up survey.
